# Perioperative and Long-Term Outcomes of Surgery in Patients Aged 80 Years or Older with Esophageal or Esophagogastric Junction Cancer: A Propensity Score–Matched Analysis

**DOI:** 10.3390/cancers18213574

**Published:** 2026-11-06

**Authors:** Takahiro Ochiai, Masanobu Nakajima, Masatoshi Nakagawa, Junki Fujita, Minori Mise, Yasunori Maeda, Masaki Yoshimatsu, Eri Amagai, Shinji Morita, Kazuyuki Kojima

**Affiliations:** 1 Department of Upper Gastrointestinal Surgery, Dokkyo Medical University, 880 Kitakobayashi, Mibu, Shimotsugagun, Tochigi 321-0293, Japan; 2 Department of Surgery, Faculty of Medicine, University of Miyazaki, 5200 Kihara, Kiyotake, Miyazaki 889-1692, Japan

**Keywords:** esophageal cancer, esophagogastric junction cancer, older patients, surgical outcomes, propensity score matching, overall survival, disease-specific survival

## Abstract

Background/Objectives: With population aging, determining appropriate surgical indications for patients aged ≥80 years with esophageal or esophagogastric junction cancer has become increasingly important. This study compared perioperative and long-term outcomes between surgically treated patients aged ≥80 years and those aged <80 years. Methods: Patients who underwent surgery for esophageal or esophagogastric junction cancer between April 2009 and April 2026 were retrospectively analyzed. Propensity score matching was performed using sex, American Society of Anesthesiologists physical status, tumor location, histological type, clinical stage, surgical procedure, and neoadjuvant chemotherapy. After matching, 40 patients were included in each group. Perioperative outcomes, postoperative complications, overall survival (OS), and disease-specific survival (DSS) were compared. Results: Operative time, blood loss, and postoperative hospital stay did not differ significantly between the groups. Patients aged ≥80 years had fewer dissected lymph nodes (26.0 vs. 48.5, *p* = 0.003) and a lower incidence of Clavien–Dindo grade ≥IIIa complications (17.5% vs. 40.0%, *p* = 0.047). The incidences of pneumonia, recurrent laryngeal nerve palsy, and anastomotic leakage did not differ significantly. The 5-year OS was lower in patients aged ≥80 years (32.9% vs. 55.3%, *p* = 0.006; HR, 2.31; 95% CI, 1.24–4.28), whereas 5-year DSS did not differ significantly (55.4% vs. 69.0%, *p* = 0.122; HR, 1.86; 95% CI, 0.84–4.12). Conclusions: Surgery may be feasible in carefully selected patients aged ≥80 years. These findings do not establish a survival advantage over non-surgical treatment. Surgical eligibility should be based on overall condition, oncological factors, and anticipated surgical invasiveness rather than chronological age alone.

## Simple Summary

Choosing appropriate surgical treatment for patients aged 80 years or older with esophageal or esophagogastric junction cancer is important in aging populations. We retrospectively compared surgical outcomes between older and younger patients after matching them to reduce differences in patient, cancer, and treatment characteristics. Each group included 40 patients. Older patients had fewer lymph nodes removed and fewer severe postoperative complications. The rates of pneumonia, vocal cord paralysis, and leakage at the surgical connection did not differ significantly. Overall survival was poorer in older patients, whereas survival considering only deaths from esophageal or esophagogastric junction cancers did not differ significantly. These findings suggest that surgery may be feasible in carefully selected patients aged 80 years or older, but they do not establish superiority over non-surgical treatment. Surgical eligibility should therefore consider the patient’s overall condition, tumor characteristics, and anticipated surgical invasiveness rather than age alone.

## 1. Introduction

With the aging of the population, determining appropriate treatment strategies for older patients with esophageal or esophagogastric junction (EGJ) cancer has become an increasingly important clinical issue. Surgical resection remains an important curative treatment option for resectable esophageal and EGJ cancer [1,2]. Depending on tumor location and extent, surgical procedures for EGJ cancer may include esophagectomy, proximal gastrectomy, or total gastrectomy [2,3]. Esophagectomy, in particular, is a highly invasive procedure associated with substantial postoperative morbidity and perioperative mortality [4,5].

The safety and long-term outcomes of surgical treatment for esophageal cancer in older patients have been extensively investigated, but the findings remain inconsistent. Older patients have been reported to have higher risks of 90-day and long-term mortality after esophagectomy, as well as an increased incidence of postoperative complications [6,7]. In contrast, Kanda et al. reported in a propensity score–matched analysis of patients aged 75 years or older that appropriately selected older patients did not have significantly higher rates of postoperative complications or surgery-related mortality, and that no significant differences were observed in overall survival (OS), disease-free survival (DFS), or disease-specific survival (DSS) [8]. Recent propensity score–matched studies have also reported no substantial differences in perioperative outcomes between older and younger patients, although long-term OS may be poorer in older patients [9,10,11].

Surgical treatment in patients aged 80 years or older requires particular consideration of both its potential benefits and perioperative risks. Motoyama et al. reported that not all patients aged 80 years or older derive a survival benefit from esophagectomy and emphasized the importance of patient selection based not only on chronological age but also on overall health status [12]. In addition, patients aged 80 years or older have been reported to have poorer OS and higher perioperative mortality and overall postoperative morbidity, whereas no clear differences have been observed in specific postoperative complications such as anastomotic leakage, respiratory complications, and recurrent laryngeal nerve injury [13]. Furthermore, a study including patients aged 80 years or older with EGJ cancer suggested that curative surgical treatment is feasible in carefully selected patients [14], while a study of curative resection for gastric and EGJ adenocarcinoma, including gastrectomy, found no significant increase in the incidence or severity of 90-day postoperative complications in patients aged 80 years or older compared with younger patients [15]. However, propensity score–matched studies specifically focusing on patients aged ≥80 years remain limited. Therefore, we selected 80 years as the age threshold to specifically evaluate surgical outcomes in this understudied population. Because EGJ cancer may require either esophagectomy or gastrectomy depending on tumor location and extent, both esophageal and EGJ cancers were included to reflect the spectrum of surgical treatment encountered in clinical practice. The primary objective of this study was to compare perioperative and long-term outcomes between surgically treated patients aged ≥80 years and those aged <80 years with esophageal or EGJ cancer using propensity score matching (PSM).

## 2. Materials and Methods

### 2.1. Patients and Study Design

**Figure 1:**
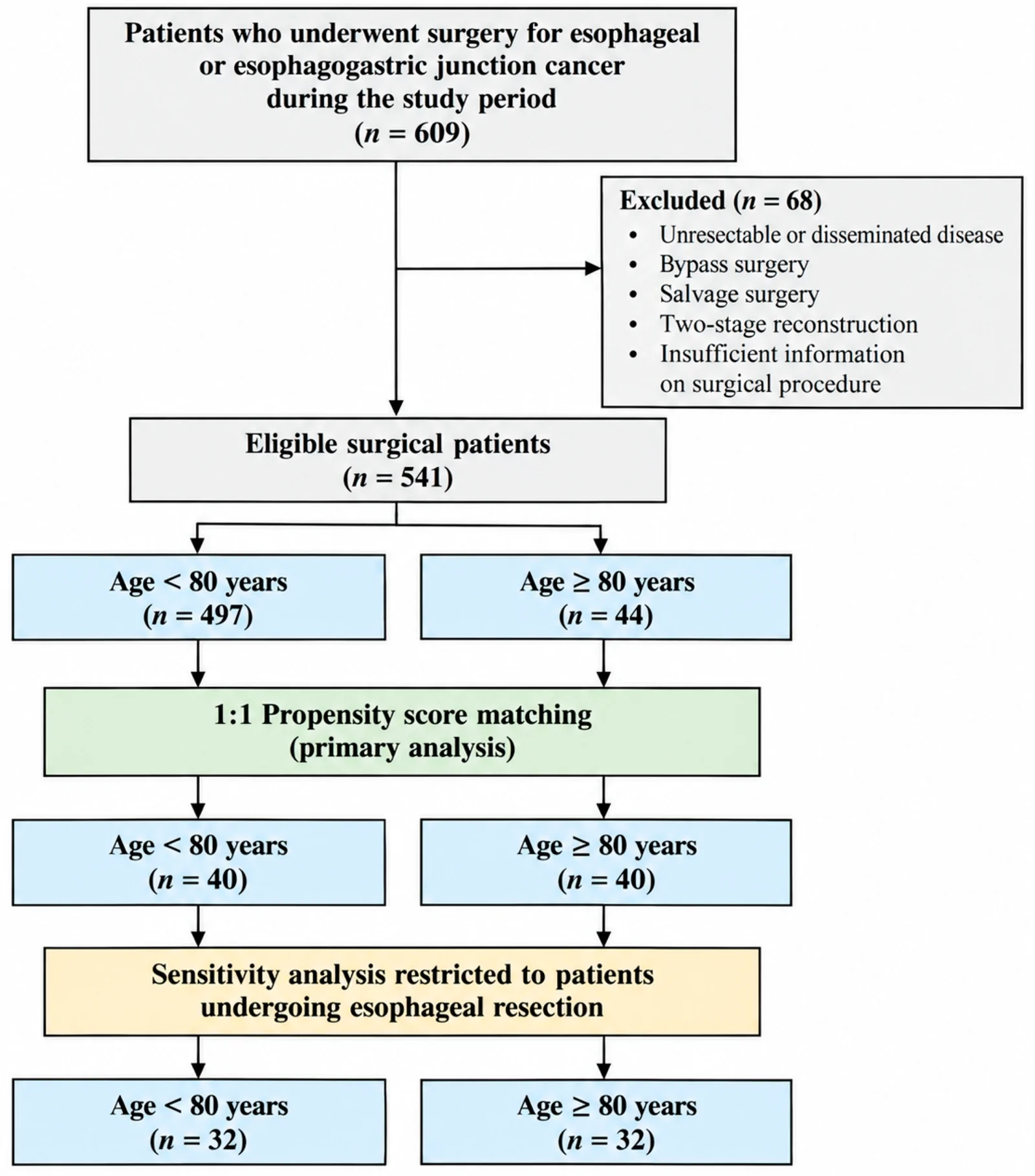
Flow diagram of patient selection and propensity score matching.

A total of 609 patients underwent surgery for esophageal or EGJ cancer at our institution between April 2009 and April 2026. After excluding 68 patients with unresectable or disseminated disease, those undergoing bypass surgery, salvage surgery, or two-stage reconstruction, and those with insufficient information on the surgical procedure, 541 patients were included in the eligible cohort (Figure 1). The study population included patients who underwent surgery as initial treatment, those who underwent planned surgery after neoadjuvant chemotherapy (NAC), and those who underwent additional surgical resection after noncurative endoscopic resection.

Patients were classified into two groups according to age at surgery: those aged 80 years or older and those younger than 80 years. EGJ cancer was defined according to the Nishi classification. For patients with esophagogastric junction cancer, the surgical procedure was selected according to tumor location and extent, with either esophagectomy or gastrectomy performed as appropriate. Esophagectomy comprised thoracoscopic, robotic, open, mediastinoscopic, and transhiatal approaches, as well as pharyngolaryngoesophagectomy. In patients aged ≥80 years, surgical candidacy was determined individually based on ASA-PS, comorbidities, cardiopulmonary function, nutritional status, tumor resectability and extent, anticipated surgical invasiveness, and patient preference. Predicted perioperative risk was also reviewed using the Japanese National Clinical Database (NCD) risk calculator as supportive information for surgical decision-making. No single physiological parameter or comorbidity score was used as an absolute criterion for surgical eligibility. The surgical approach was selected according to the patient’s physiological reserve and tumor characteristics. In patients with reduced pulmonary reserve, particularly those with a forced expiratory volume in 1 s (FEV1) of around or below 1.5 L, mediastinoscopic esophagectomy was preferentially considered to reduce thoracic surgical invasiveness. To provide additional context regarding treatment selection in older patients, patients aged ≥80 years who received definitive chemoradiotherapy during the same study period were also reviewed descriptively. These patients were not included in the primary comparative or propensity score–matched analyses.

This study was approved by the Medical Ethics Committee of Dokkyo Medical University (approval no. R-110-1J).

### 2.2. Clinicopathological Characteristics and Outcomes

Patient and tumor characteristics included age, sex, American Society of Anesthesiologists physical status (ASA-PS), tumor location, histological type, clinical stage, surgical procedure, and receipt of NAC. In the matched cohort, the Charlson Comorbidity Index (CCI), body mass index (BMI), and prognostic nutritional index (PNI) were also evaluated. The PNI was calculated using the most recent preoperative laboratory results. The CCI was assessed based on the type and severity of comorbidities, and the proportion of patients with CCI ≥ 2 was compared between the groups. Formal frailty, cognitive, and functional assessment tools were not routinely used throughout the study period and were therefore not included in the analysis. For patients undergoing esophagectomy, the extent of lymphadenectomy was categorized as two-field, three-field, or limited lymphadenectomy. Limited lymphadenectomy included cases in which upper mediastinal lymph node dissection was omitted or restricted, as well as transhiatal esophagectomy in which lymphadenectomy was generally limited to the abdominal field without systematic mediastinal lymph node dissection. The extent of lymphadenectomy was selected according to tumor location, disease extent, surgical approach, and the patient’s overall condition, in accordance with the Japanese Esophageal Society guidelines. For patients undergoing gastrectomy, lymphadenectomy was categorized as D1+ or D2 according to the Japanese Gastric Cancer Association guidelines. Surgical and pathological outcomes included operative time, blood loss, the number of dissected and metastatic lymph nodes, pathological stage, R0 resection rate, and postoperative length of hospital stay. Postoperative complications occurring within 30 days after surgery were graded according to the Clavien–Dindo classification, and the incidence of grade IIIa or higher complications was compared between the groups. Pneumonia, anastomotic leakage, and recurrent laryngeal nerve palsy of grade I or higher were evaluated separately. Recurrent laryngeal nerve palsy was assessed based on clinical symptoms, including hoarseness. 30- and 90-day postoperative mortality rates were also assessed. Long-term outcomes included OS and DSS, with survival time measured from the date of surgery and 5-year survival rates estimated for both endpoints. For DSS, deaths from causes other than esophageal or EGJ cancer were censored at the date of death.

### 2.3. Propensity Score Matching

PSM was performed to adjust for baseline differences between patients aged 80 years or older and those younger than 80 years. Propensity scores were estimated using a logistic regression model with sex, ASA-PS, tumor location, histological type, clinical stage, surgical procedure, and the use of NAC as covariates. These covariates were selected based on their clinical relevance to perioperative and oncological outcomes and their consistent availability across the study cohort. CCI, PNI, and BMI were not included in the propensity score model because of missing data in the unmatched cohort, particularly among patients aged <80 years. These variables were therefore assessed separately in the matched cohort. One-to-one nearest-neighbor matching without replacement was performed using a caliper width of 0.2 standard deviations of the logit of the propensity score. After matching, 40 patients aged 80 years or older and 40 patients younger than 80 years were included in the matched cohort. Covariate balance before and after matching was assessed using standardized mean differences (SMDs) calculated in EZR. For multicategory categorical variables, a single SMD was reported for each variable as a whole. An absolute SMD < 0.10 was considered indicative of adequate balance. As an additional sensitivity analysis to reduce heterogeneity related to surgical procedures, the cohort was restricted to patients undergoing esophageal resection. The same propensity score model and matching procedure as in the primary analysis were then applied, resulting in 32 matched patients in each age group.

### 2.4. Statistical Analysis

Categorical variables were compared using the χ^2^ test or Fisher’s exact test, as appropriate. Continuous variables were analyzed using Student’s t-test or the Mann–Whitney U test, as appropriate. No imputation was performed for missing data. Analyses were conducted using available cases, and patients with missing values for variables required for a given analysis were excluded from that analysis. Effect estimates with 95% confidence intervals were reported for the principal perioperative outcomes. For continuous variables, effect estimates were expressed as Hodges–Lehmann estimates of location shift, whereas odds ratios were used for binary variables. OS and DSS were estimated using the Kaplan–Meier method, and survival curves were compared using the log-rank test. Hazard ratios (HRs) with 95% confidence intervals were estimated using Cox proportional hazards models. As a sensitivity analysis, Cox models were additionally adjusted for ASA-PS and grouped surgical procedure/approach to account for residual imbalance after PSM. For this analysis, surgical procedures and approaches were grouped into four clinically relevant categories: thoracoscopic or robotic esophagectomy, open esophagectomy, mediastinoscopic esophagectomy or transhiatal esophagectomy, and gastrectomy. Median follow-up duration was estimated using the reverse Kaplan–Meier method, with an administrative censoring date of 7 October 2026. All statistical tests were two-sided, and a *p*-value < 0.05 was considered statistically significant.

All statistical analyses were performed using EZR version 1.68 (Saitama Medical Center, Jichi Medical University, Saitama, Japan), a graphical user interface for R version 4.3.1 (R Foundation for Statistical Computing, Vienna, Austria).

## 3. Results

### 3.1. Patient Characteristics and Propensity Score Matching

Among the 541 eligible patients, 44 were aged ≥80 years and 497 were aged <80 years. Patient characteristics before and after matching are shown in Table 1. All paired values below are presented in the order of patients aged ≥80 years versus those aged <80 years. Before matching, the proportions of patients undergoing esophagectomy (84.1% vs. 94.8%) and gastrectomy (15.9% vs. 5.2%) differed between the groups. NAC was administered less frequently in patients aged ≥80 years (18.2% vs. 31.4%).

**Figure 2:**
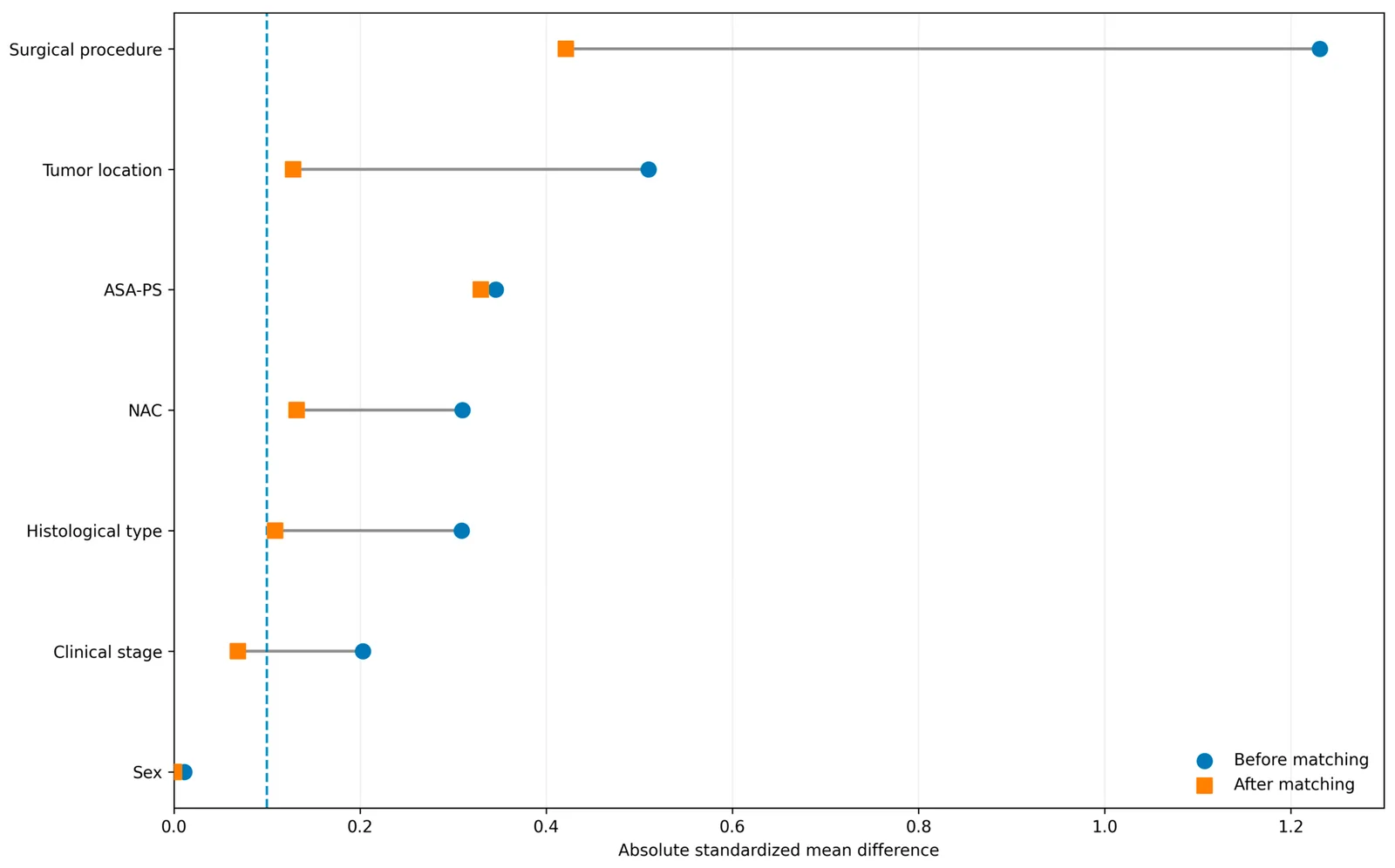
Standardized mean differences in covariates before and after propensity score matching. The dashed line indicates an absolute standardized mean difference of 0.10.

PSM resulted in 40 patients in each group. Standardized mean differences before and after PSM are shown in Figure 2. After matching, the proportions of patients undergoing esophagectomy (85.0% vs. 85.0%) and gastrectomy (15.0% vs. 15.0%) were identical between the groups. The SMDs were <0.001 for sex, 0.069 for clinical stage, 0.109 for histological type, 0.128 for tumor location, and 0.132 for NAC. Residual imbalance remained in ASA-PS (SMD = 0.330) and the distribution of specific surgical procedures and approaches (SMD = 0.421).

In the matched cohort, no significant differences were observed in the proportion of patients with CCI ≥ 2 (30.0% vs. 25.0%, *p* = 0.803), PNI (43.4 vs. 47.3, *p* = 0.122), or BMI (21.9 vs. 22.7, *p* = 0.521).

During the same study period, 35 patients aged ≥80 years received definitive chemoradiotherapy with curative intent. Treatment selection reflected the patient’s overall condition, tumor characteristics, anticipated treatment invasiveness, and patient preference.

### 3.2. Surgical Outcomes and Postoperative Complications

Surgical outcomes and postoperative complications in the matched cohort, including effect estimates with 95% confidence intervals for the principal perioperative outcomes, are summarized in Table 2. Continuous variables are presented as medians. Operative time (446 vs. 509 min, *p* = 0.272), blood loss (385 vs. 368.5 mL, *p* = 0.461), and postoperative length of hospital stay (29 vs. 23.5 days, *p* = 0.497) did not differ significantly between the groups. Three-field lymphadenectomy was less frequent, whereas limited lymphadenectomy was more frequent, in patients aged ≥80 years; however, the overall distribution of lymphadenectomy extent did not differ significantly between the groups (*p* = 0.095).

The number of dissected lymph nodes was significantly lower in patients aged ≥80 years (26.0 vs. 48.5, *p* = 0.003). In contrast, no significant differences were observed in the number of metastatic lymph nodes (1.0 vs. 1.0, *p* = 0.980), pathological stage (*p* = 0.441), or R0 resection rate (87.5% vs. 95.0%, *p* = 0.432).

The incidence of postoperative complications of Clavien–Dindo grade IIIa or higher was significantly lower in patients aged ≥80 years (17.5% vs. 40.0%, *p* = 0.047). The incidences of pneumonia (10.0% vs. 7.5%, *p* = 1.000), recurrent laryngeal nerve palsy (12.5% vs. 7.5%, *p* = 0.712), and anastomotic leakage (17.5% vs. 30.0%, *p* = 0.293) did not differ significantly between the groups. Neither 30-day nor 90-day postoperative mortality differed significantly between the groups (0% vs. 2.5% for both outcomes, both *p* = 1.000).

### 3.3. Long-Term Outcomes

**Figure 3:**
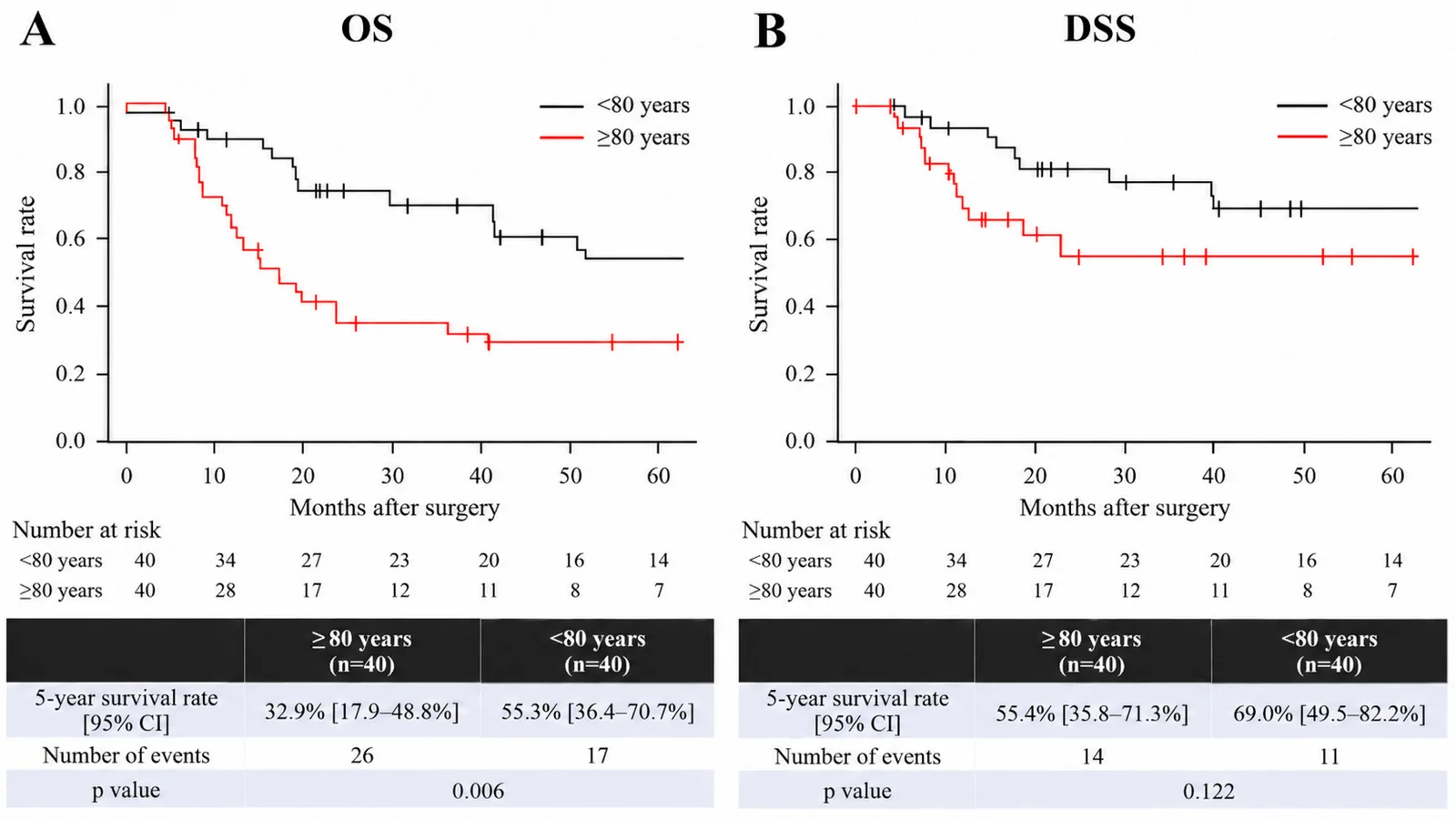
Kaplan–Meier survival curves after propensity score matching. (**A**) Overall survival. (**B**) Disease-specific survival.

Kaplan–Meier survival curves for OS and DSS are shown in Figure 3A and Figure 3B, respectively. The 5-year OS rate was significantly lower in patients aged ≥80 years than in those aged <80 years (32.9% [95% CI, 17.9–48.8%] vs. 55.3% [95% CI, 36.4–70.7%], *p* = 0.006). Cox proportional hazards analysis showed a significantly higher hazard of all-cause mortality in patients aged ≥80 years (HR, 2.31; 95% CI, 1.24–4.28; *p* = 0.008). The 5-year DSS rate did not differ significantly between the groups (55.4% [95% CI, 35.8–71.3%] vs. 69.0% [95% CI, 49.5–82.2%], *p* = 0.122). The corresponding HR for disease-specific mortality was 1.86 (95% CI, 0.84–4.12; *p* = 0.127). In sensitivity analyses adjusting for ASA-PS and grouped surgical procedure/approach, the association between age ≥80 years and poorer OS remained materially unchanged (adjusted HR, 2.43; 95% CI, 1.29–4.58; *p* = 0.006), whereas the association with DSS remained non-significant (adjusted HR, 2.03; 95% CI, 0.90–4.59; *p* = 0.089). During follow-up, 26 deaths occurred in patients aged ≥80 years and 17 in those aged <80 years. Of these, 14 and 11 deaths, respectively, were disease-specific, whereas 11 and 6 were due to causes other than esophageal or EGJ cancer. The cause of death was unknown in one patient aged ≥80 years. Pneumonia was the most frequent cause of death other than esophageal or EGJ cancer in patients aged ≥80 years (*n* = 6). The median follow-up duration estimated using the reverse Kaplan–Meier method was 61.4 months overall, 57.1 months in patients aged ≥80 years, and 61.5 months in those aged <80 years.

### 3.4. Sensitivity Analysis Restricted to Patients Undergoing Esophageal Resection

**Figure 4:**
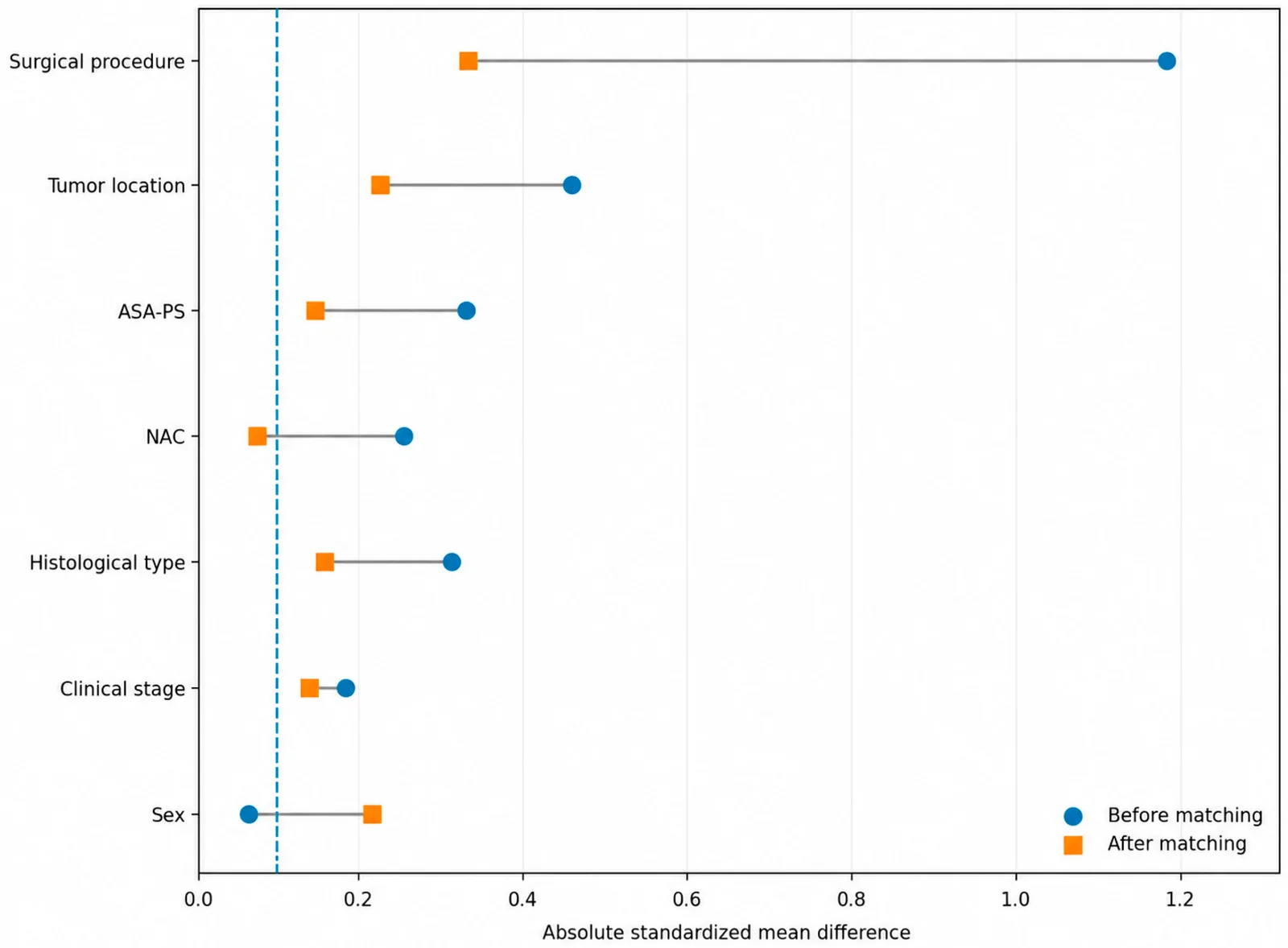
Standardized mean differences in covariates before and after propensity score matching in the esophagectomy-only cohort. The dashed line indicates an absolute standardized mean difference of 0.10.

As a sensitivity analysis, the cohort was restricted to patients undergoing esophageal resection. After matching, 32 patients were included in each age group (Table 3). Covariate balance before and after propensity score matching in this esophagectomy-only cohort is shown in Figure 4. Residual imbalance remained in several covariates, particularly surgical procedure/approach (SMD = 0.334), tumor location (SMD = 0.225), and sex (SMD = 0.216).

Surgical and postoperative outcomes are summarized in Table 4. Operative time (424.0 vs. 494.0 min, *p* = 0.213), blood loss (378.5 vs. 312.5 mL, *p* = 0.204), and postoperative hospital stay (29.0 vs. 22.0 days, *p* = 0.368) did not differ significantly between patients aged ≥80 years and those aged <80 years. Three-field lymphadenectomy was less frequent and limited lymphadenectomy was more frequent in patients aged ≥80 years, although the overall distribution of lymphadenectomy extent did not differ significantly between the groups (*p* = 0.079). The number of dissected lymph nodes remained significantly lower in patients aged ≥80 years (25.0 vs. 40.5, *p* = 0.010), whereas the number of metastatic lymph nodes (1.0 vs. 1.0, *p* = 0.831), pathological stage (*p* = 0.603), and R0 resection rate (90.6% vs. 90.6%, *p* = 1.000) did not differ significantly between the groups. The incidence of Clavien–Dindo grade ≥IIIa complications was lower in patients aged ≥80 years, although the difference was not statistically significant (21.9% vs. 46.9%, *p* = 0.064). No significant differences were observed in pneumonia, recurrent laryngeal nerve palsy, anastomotic leakage, or 30- or 90-day mortality.

**Figure 5:**
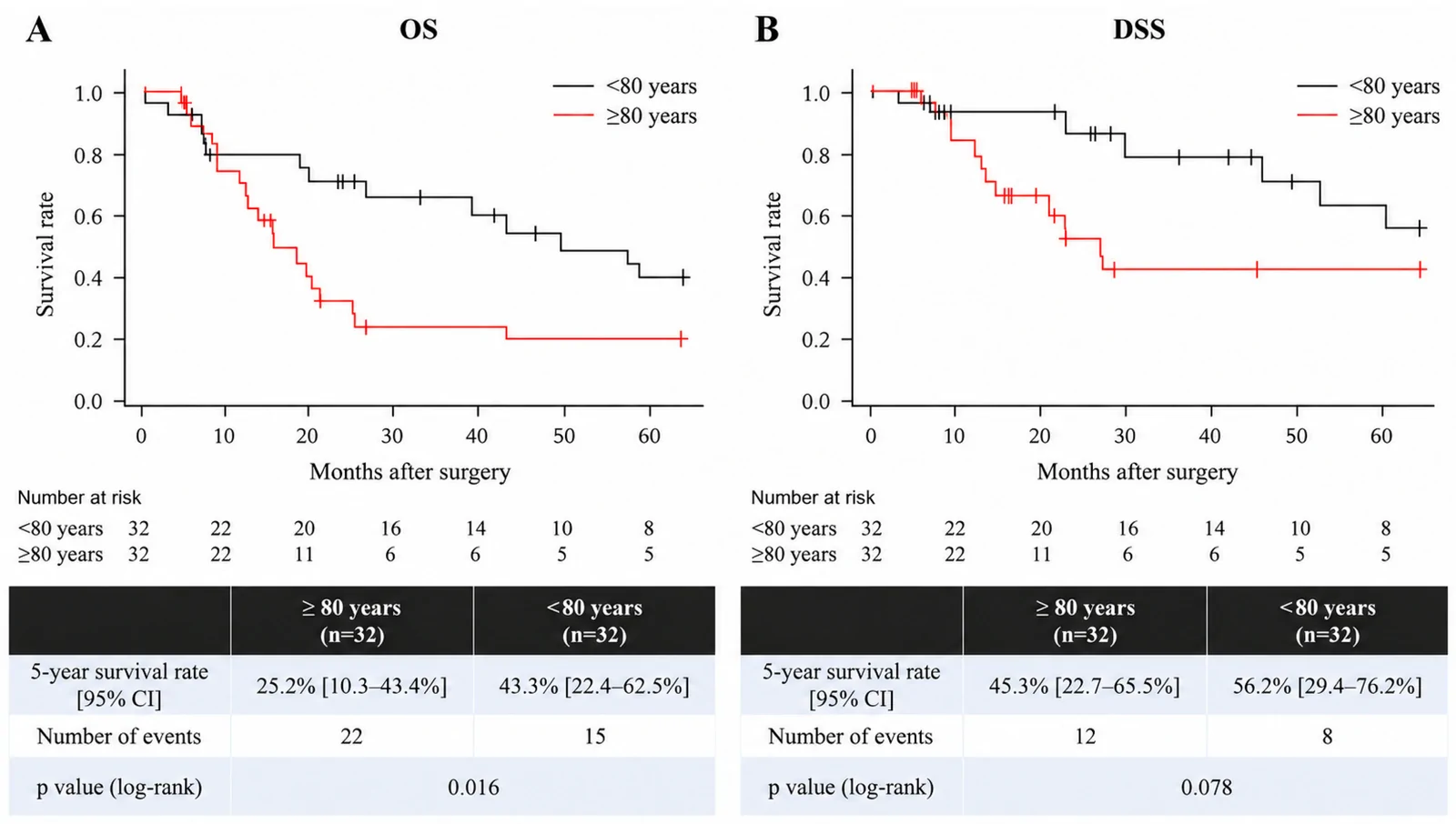
Kaplan–Meier curves for overall survival (**A**) and disease-specific survival (**B**) in the esophagectomy-only matched cohort.

Kaplan–Meier curves for OS and DSS in the esophagectomy-only matched cohort are shown in Figure 5A and Figure 5B, respectively. The 5-year OS rate was significantly lower in patients aged ≥80 years than in those aged <80 years (25.2% [95% CI, 10.3–43.4%] vs. 43.3% [95% CI, 22.4–62.5%], *p* = 0.016). The corresponding HR for all-cause mortality was 2.24 (95% CI, 1.14–4.37; *p* = 0.019). The 5-year DSS rate was 45.3% (95% CI, 22.7–65.5%) in patients aged ≥80 years and 56.2% (95% CI, 29.4–76.2%) in those aged <80 years, with no statistically significant difference between the groups (*p* = 0.078). The corresponding HR for disease-specific mortality was 2.22 (95% CI, 0.89–5.52; *p* = 0.085). The median follow-up duration estimated using the reverse Kaplan–Meier method was 61.4 months overall, 61.4 months in patients aged ≥80 years, and 62.1 months in those aged <80 years.

## 4. Discussion

Although surgical outcomes in older patients with esophageal cancer have been widely investigated, propensity score–matched comparisons specifically focusing on patients aged ≥80 years remain limited [13]. In addition to patients undergoing esophagectomy for esophageal cancer, the present study included patients undergoing esophagectomy or gastrectomy for EGJ cancer. Using PSM, we compared perioperative and long-term outcomes between patients aged ≥80 years and those aged <80 years. After matching, patients aged ≥80 years did not have higher rates of major postoperative complications or perioperative mortality. Long-term OS was significantly poorer in this group, whereas DSS did not differ significantly between the groups. These findings suggest that surgical outcomes in patients aged ≥80 years should be evaluated in light of patient selection, surgical invasiveness, and deaths from other causes, in addition to chronological age.

Regarding perioperative outcomes, previous studies have reported no significant differences in postoperative complications or perioperative mortality between carefully selected older patients and younger patients undergoing surgery for esophageal cancer [9,11,16,17,18]. No significant increases in specific complications, including anastomotic leakage, respiratory complications, and recurrent laryngeal nerve injury, have been reported in patients aged ≥80 years [13]. Similarly, in the present study, patients aged ≥80 years did not have significantly higher rates of major postoperative complications, including pneumonia and anastomotic leakage, and the incidence of recurrent laryngeal nerve palsy did not differ significantly between the groups. Furthermore, no 30-day or 90-day postoperative deaths occurred in patients aged ≥80 years, and the incidence of severe postoperative complications of Clavien–Dindo grade ≥IIIa was significantly lower than in patients aged <80 years.

Patients aged ≥80 years in this study were carefully selected for surgery based on their overall condition, organ reserve, tumor extent, and anticipated surgical invasiveness; therefore, the influence of patient selection should be considered. ASA-PS was included as a covariate in PSM, and CCI, PNI, and BMI did not differ significantly between the groups after matching. However, residual imbalance remained in ASA-PS and the distribution of specific surgical procedures and approaches after PSM. In addition, unmeasured factors or residual confounding may have contributed to the difference in severe postoperative complications. In particular, patients aged ≥80 years who ultimately underwent surgery may have represented a relatively fit group with preserved physical and cognitive function and lower levels of frailty. Such selection may have contributed to the lower incidence of severe postoperative complications observed in the older group. Scarpa et al. reported that age itself was not a significant predictor of postoperative complications in older patients with esophageal cancer, whereas the age-adjusted Charlson Comorbidity Index was useful for assessing fitness for surgery and PNI for predicting postoperative complications [19]. Associations between frailty and postoperative mortality or major complications have also been demonstrated in meta-analyses of patients undergoing esophagectomy and older patients with gastroesophageal cancer, as well as in PSM analyses [20,21,22]. Therefore, surgical eligibility in older patients should be determined through an individualized assessment of comorbidities, nutritional status, frailty, physical function, oncological factors, and anticipated surgical invasiveness, in addition to chronological age.

Another notable finding of this study was the significantly lower number of dissected lymph nodes in patients aged ≥80 years. In contrast, the number of metastatic lymph nodes, pathological stage, and R0 resection rate did not differ significantly between the groups. Although the overall distribution of lymphadenectomy extent did not differ significantly between the groups, three-field lymphadenectomy was less frequent and limited lymphadenectomy was more frequent in patients aged ≥80 years in both the primary matched cohort and the esophagectomy-only matched cohort. These descriptive differences in lymphadenectomy extent may have contributed, at least in part, to the lower lymph node yield observed in older patients. The lower lymph node yield persisted in the sensitivity analysis restricted to patients undergoing esophageal resection (25.0 vs. 40.5, *p* = 0.010), indicating that the difference was not attributable solely to the inclusion of different types of surgical resection in the primary analysis. However, differences in lymphadenectomy extent and residual imbalance in surgical procedure/approach and tumor location remained in the restricted analysis; therefore, the respective contributions of age, tumor location, surgical approach, and lymphadenectomy extent to the lower lymph node yield could not be clearly separated.

Within guideline-based practice, surgical invasiveness, including the extent of lymphadenectomy and the surgical procedure, may have been tailored to individual older patients based on comorbidities and organ reserve. Previous studies have reported more limited mediastinal lymphadenectomy in older patients and a decrease in the use of three-field lymphadenectomy with increasing age [23,24]. However, a meta-analysis reported a significantly lower R0 resection rate in octogenarians undergoing esophagectomy [13], highlighting the need to balance surgical invasiveness and oncological radicality in older patients. Despite the absence of statistically significant differences in pathological stage and R0 resection rate, the possibility of stage migration associated with the lower lymph node yield cannot be completely excluded. The present study cannot determine the effect of fewer dissected lymph nodes on long-term oncological outcomes. Regarding short-term outcomes, a study of patients aged ≥80 years with thoracic esophageal cancer reported no significant differences in 30-day, 60-day, or 90-day mortality between patients with <15 and ≥15 dissected lymph nodes [25]. As the association between the number of dissected lymph nodes and severe postoperative complications was not directly examined in the present study, the lower incidence of Clavien–Dindo grade ≥IIIa complications in the older group should not be attributed to less extensive lymphadenectomy.

Multiple factors, including patient selection, surgical procedures, and perioperative management, may have contributed to the lower incidence of severe postoperative complications observed in this study. Further investigation is needed to determine the appropriate extent of lymphadenectomy in older patients.

It should also be noted that this study included patients with EGJ cancer, for whom different surgical procedures, including esophagectomy and gastrectomy, were selected according to tumor location and extent. Studies of older patients, including those with EGJ cancer, have suggested that surgical resection may be a treatment option in carefully selected patients [14,15,26,27]. However, a population-based study of esophagogastric cancer, including esophageal and gastric cancer, demonstrated that increasing age was associated with lower rates of surgical treatment and a higher risk of long-term mortality after surgery [28]. Although surgical procedure was included as a covariate in PSM in the present study, differences in surgical procedures and the extent of lymphadenectomy for EGJ cancer may have influenced the number of dissected lymph nodes and perioperative outcomes. This heterogeneity should therefore be considered when interpreting the results.

Regarding long-term outcomes, OS was significantly poorer in patients aged ≥80 years than in those aged <80 years in the present study, whereas DSS did not differ significantly between the groups. A systematic review and meta-analysis also reported significantly poorer OS in octogenarians than in non-octogenarians undergoing curative esophagectomy for esophageal cancer [13]. Previous studies of older patients with esophageal cancer have reported poorer OS or a trend toward poorer OS, without significant differences in cancer-specific survival [18,29]. Another study reported that older age was associated with poorer OS but not with DFS after adjustment [30]. The divergence between OS and DSS observed in the present study may partly reflect the greater contribution of deaths from causes other than esophageal or EGJ cancer in patients aged ≥80 years, particularly deaths due to pneumonia. Previous studies have also reported that pneumonia, impaired pulmonary function, comorbidity burden, and sarcopenia are associated with non-cancer mortality after esophagectomy [31,32,33,34]. Motoori et al. reported that age was not a significant prognostic factor for long-term survival after esophagectomy in an analysis accounting for deaths unrelated to cancer [35]. Taken together, these findings suggest that long-term outcomes in older patients should be evaluated by considering not only cancer-specific outcomes but also competing mortality, and that postoperative follow-up should include attention to respiratory and nutritional deterioration in addition to cancer recurrence.

This study has several limitations. First, it was a single-center retrospective study. The primary analysis included only patients who underwent surgery. Although 35 patients aged ≥80 years received definitive chemoradiotherapy during the study period, treatment allocation was not randomized and reflected differences in patient condition, tumor characteristics, anticipated treatment invasiveness, and patient preference. Therefore, the present study cannot determine whether surgery provides a survival advantage over definitive chemoradiotherapy or establish a validated algorithm for selecting older patients for surgery. Although PSM was used to adjust for measured background factors, residual confounding by unmeasured factors, such as frailty, cannot be fully excluded. In particular, formal frailty, cognitive, and functional assessments were not routinely performed throughout the study period, and their potential influence on treatment selection and outcomes could not be evaluated. Residual imbalance in ASA-PS and the distribution of specific surgical procedures and approaches after PSM may also have influenced comparisons of perioperative and long-term outcomes. Second, the matched cohort was limited to 40 patients in each group, which may have provided insufficient statistical power to detect differences in DSS and outcomes with few events, such as 30-day and 90-day mortality. Third, the long study period encompassed changes in surgical techniques and perioperative management, and potential era effects cannot be fully excluded. The precision of long-term survival estimates, particularly 5-year survival rates, should also be interpreted cautiously because of the limited sample size and the decreasing number of patients at risk over time.

Prospective multicenter studies assessing factors particularly relevant to older patients, such as frailty, are needed to stratify both perioperative risk and long-term prognosis, including non-cancer mortality. Such studies may help identify indicators to guide appropriate patient selection for surgery and tailor the invasiveness of surgery to individual patients aged ≥80 years.

## 5. Conclusions

Surgical treatment may be feasible in carefully selected surgical candidates aged ≥80 years with esophageal or EGJ cancer. However, the potential influence of competing mortality from causes other than esophageal or EGJ cancer should be considered when evaluating long-term outcomes. These findings should not be interpreted as demonstrating a survival advantage of surgery over non-surgical treatment. Therefore, surgical eligibility should be determined through a comprehensive assessment of the patient’s overall condition, oncological factors, and anticipated surgical invasiveness, rather than chronological age alone.

## Figures and Tables

**Table 1:** Patient characteristics before and after propensity score matching.

Variable	Before Matching			After Matching		
	≥80 Years (*n* = 44)	<80 Years (*n* = 497)	SMD	≥80 Years (*n* = 40)	<80 Years (*n* = 40)	SMD
Age in years, median (IQR)	83 (81–84)	68 (61–73)	—	83 (81–84)	71 (64–75)	—
Sex, *n* (%)			0.011			<0.001
Male	37 (84.1)	420 (84.5)		35 (87.5)	35 (87.5)	
Female	7 (15.9)	77 (15.5)		5 (12.5)	5 (12.5)	
ASA-PS, *n* (%)			0.346			0.330
1	0 (0.0)	26 (5.2)		0 (0.0)	1 (2.5)	
2	42 (95.5)	458 (92.2)		39 (97.5)	36 (90.0)	
3	2 (4.5)	13 (2.6)		1 (2.5)	3 (7.5)	
Tumor location, *n* (%)			0.510			0.128
Ce	0 (0.0)	21 (4.2)		0 (0.0)	0 (0.0)	
Ut	4 (9.1)	60 (12.1)		4 (10.0)	4 (10.0)	
Mt	11 (25.0)	195 (39.2)		10 (25.0)	10 (25.0)	
Lt	19 (43.2)	155 (31.2)		17 (42.5)	15 (37.5)	
Jz	10 (22.7)	66 (13.3)		9 (22.5)	11 (27.5)	
Histological type, *n* (%)			0.309			0.109
SCC	32 (72.7)	396 (79.7)		29 (72.5)	27 (67.5)	
Adenocarcinoma	12 (27.3)	89 (17.9)		11 (27.5)	13 (32.5)	
Others	0 (0.0)	12 (2.4)		0 (0.0)	0 (0.0)	
Clinical stage, *n* (%)			0.203			0.069
0	1 (2.3)	21 (4.2)		1 (2.5)	1 (2.5)	
I	10 (22.7)	139 (28.0)		8 (20.0)	9 (22.5)	
II	15 (34.1)	137 (27.6)		14 (35.0)	14 (35.0)	
III	15 (34.1)	158 (31.9)		14 (35.0)	13 (32.5)	
IV	3 (6.8)	41 (8.3)		3 (7.5)	3 (7.5)	
Surgical procedure, *n* (%)			1.231			0.421
Esophagectomy						
Open	9 (20.5)	136 (27.4)		9 (22.5)	12 (30.0)	
Thoracoscopic	6 (13.6)	240 (48.3)		6 (15.0)	8 (20.0)	
Robotic	4 (9.1)	51 (10.3)		4 (10.0)	3 (7.5)	
Mediastinoscopic	8 (18.2)	14 (2.8)		8 (20.0)	4 (10.0)	
Transhiatal esophagectomy	10 (22.7)	20 (4.0)		7 (17.5)	7 (17.5)	
Pharyngolaryngoesophagectomy	0 (0.0)	10 (2.0)		0 (0.0)	0 (0.0)	
Gastrectomy						
Open	3 (6.8)	16 (3.2)		3 (7.5)	2 (5.0)	
Laparoscopic	0 (0.0)	4 (0.8)		0 (0.0)	1 (2.5)	
Robotic	4 (9.1)	6 (1.2)		3 (7.5)	3 (7.5)	
NAC, *n* (%)	8 (18.2)	156 (31.4)	0.310	8 (20.0)	6 (15.0)	0.132
Additional characteristics after matching						*p* value
CCI ≥ 2, *n* (%)	—	—	—	12 (30.0)	10 (25.0)	0.803
PNI, median (IQR)	—	—	—	43.4 (40.5–47.5)	47.3 (41.4–50.6)	0.122
BMI (kg/m^2^), median (IQR)	—	—	—	21.9 (20.0–24.0)	22.7 (20.1–25.2)	0.521

ASA-PS, American Society of Anesthesiologists Physical Status; BMI, body mass index; IQR, interquartile range; CCI, Charlson Comorbidity Index; Ce, cervical esophagus; Ut, upper thoracic esophagus; Mt, middle thoracic esophagus; Lt, lower thoracic esophagus; Jz, esophagogastric junction; NAC, neoadjuvant chemotherapy; PNI, prognostic nutritional index; PSM, propensity score matching; SCC, squamous cell carcinoma; SMD, standardized mean difference.

**Table 2:** Surgical and postoperative outcomes after propensity score matching.

Variable	≥80 Years (*n* = 40)	<80 Years (*n* = 40)	Effect Estimate (95% CI)	*p* Value
Operative time (min), median (IQR)	446.0 (365.3–553.0)	509.0 (374.8–615.0)	−51 (−126 to 26)	0.272
Blood loss (ml), median (IQR)	385.0 (213.5–576.8)	368.5 (192.5–649.3)	−21 (−165 to 106)	0.461
Postoperative hospital stay(day), median (IQR)	29.0 (20.8–43.0)	23.5 (15.0–42.5)	4 (−1 to 11)	0.497
Extent of lymphadenectomy, *n* (%)			—	0.095
Two-field	7(17.5)	4 (10.0)		
Three-field	10 (25.0)	21 (52.5)		
Limited lymphadenectomy	17 (42.5)	9 (22.5)		
D1+	5 (12.5)	5 (12.5)		
D2	1 (2.5)	1 (2.5)		
Number of dissected lymph nodes, median (IQR)	26.0 (14.8–38.0)	48.5 (20.3–74.8)	−16 (−35 to −3)	0.003
Number of metastatic lymph nodes, median (IQR)	1.0 (0.0–4.3)	1.0 (0.0–3.0)	—	0.980
Pathological stage, *n* (%)			—	0.441
0	3 (7.5)	6 (15.0)		
I	7 (17.5)	7 (17.5)		
II	10 (25.0)	9 (22.5)		
III	17 (42.5)	11 (27.5)		
IV	3 (7.5)	7 (17.5)		
R0 resection, *n* (%)	35 (87.5)	38 (95.0)	0.37 (0.03–2.46)	0.432
Clavien–Dindo grade ≥IIIa complications, *n* (%)	7 (17.5)	16 (40.0)	0.32 (0.10–0.99)	0.047
Pneumonia, *n* (%)	4 (10.0)	3 (7.5)	1.37 (0.21–9.98)	1.000
Recurrent laryngeal nerve palsy, *n* (%)	5 (12.5)	3 (7.5)	1.75 (0.31–12.11)	0.712
Anastomotic leakage, *n* (%)	7 (17.5)	12 (30.0)	0.50 (0.15–1.60)	0.293
30-day mortality, *n* (%)	0 (0.0)	1 (2.5)	—	1.000
90-day mortality, *n* (%)	0 (0.0)	1 (2.5)	—	1.000

IQR, interquartile range.

**Table 3:** Patient characteristics before and after propensity score matching in the esophagectomy-only cohort.

Variable	Before Matching			After Matching		
	≥80 Years (*n* = 37)	<80 Years (*n* = 471)	SMD	≥80 Years (*n* = 32)	<80 Years (*n* = 32)	SMD
Age in years, median (IQR)	83 (81–85)	68 (61–73)	—	83 (81–84)	72 (67–73)	—
Sex, *n* (%)			0.062			0.216
Male	32 (86.5)	397 (84.3)		28 (87.5)	30 (93.8)	
Female	5 (13.5)	74 (15.7)		4 (12.5)	2 (6.2)	
ASA-PS, *n* (%)			0.328			0.148
1	0 (0.0)	24 (5.1)		0 (0.0)	0 (0.0)	
2	36 (97.3)	436 (92.6)		31 (96.9)	30 (93.8)	
3	1 (2.7)	11 (2.3)		1 (3.1)	2 (6.2)	
Tumor location, *n* (%)			0.460			0.225
Ce	0 (0.0)	21 (4.5)		0 (0.0)	0 (0.0)	
Ut	4 (10.8)	60 (12.7)		4 (12.5)	6 (18.8)	
Mt	11 (29.7)	194 (41.2)		10 (31.2)	8 (25.0)	
Lt	18 (48.6)	150 (31.8)		15 (46.9)	14 (43.8)	
Jz	4 (10.8)	46 (9.8)		3 (9.4)	4 (12.5)	
Histological type, *n* (%)			0.314			0.161
SCC	28 (75.7)	389 (82.6)		25 (78.1)	27 (84.4)	
Adenocarcinoma	9 (24.3)	71 (15.1)		7 (21.9)	5 (15.6)	
Others	0 (0.0)	11 (2.3)		0 (0.0)	0 (0.0)	
Clinical stage, *n* (%)			0.182			0.139
0	1 (2.7)	19 (4.0)		1 (3.1)	1 (3.1)	
I	8 (21.6)	125 (26.5)		7 (21.9)	6 (18.8)	
II	13 (35.1)	131 (27.8)		11 (34.4)	10 (31.2)	
III	12 (32.4)	155 (32.9)		10 (31.2)	12 (37.5)	
IV	3 (8.1)	41 (8.7)		3 (9.4)	3 (9.4)	
Surgical procedure, *n* (%)			1.173			0.334
Esophagectomy						
Open	9 (24.3)	136 (28.9)		9 (28.1)	11 (34.4)	
Thoracoscopic	6 (16.2)	240 (51.0)		6 (18.8)	4 (12.5)	
Robotic	4 (10.8)	51 (10.8)		4 (12.5)	5 (15.6)	
Mediastinoscopic	8 (21.6)	14 (3.0)		5 (15.6)	7 (21.9)	
Transhiatal esophagectomy	10 (27.0)	20 (4.2)		8 (25.0)	5 (15.6)	
Pharyngolaryngoesophagectomy	0 (0.0)	10 (2.1)		0 (0.0)	0 (0.0)	
NAC, *n* (%)	8 (21.6)	155 (32.9)	0.256	8 (25.0)	7 (21.9)	0.074
Additional characteristics after matching						*p* value
CCI ≥ 2, *n* (%)	—	—	—	9 (28.1)	11 (34.4)	0.788
PNI, median (IQR)	—	—	—	43.3 (40.3–48.1)	46.8 (43.3–50.4)	0.036
BMI (kg/m^2^), median (IQR)	—	—	—	21.6 (19.9–23.0)	22.5 (19.7–24.6)	0.676

ASA-PS, American Society of Anesthesiologists Physical Status; BMI, body mass index; IQR, interquartile range; CCI, Charlson Comorbidity Index; Ce, cervical esophagus; Ut, upper thoracic esophagus; Mt, middle thoracic esophagus; Lt, lower thoracic esophagus; Jz, esophagogastric junction; NAC, neoadjuvant chemotherapy; PNI, prognostic nutritional index; PSM, propensity score matching; SCC, squamous cell carcinoma; SMD, standardized mean difference.

**Table 4:** Surgical and postoperative outcomes after propensity score matching in the esophagectomy-only cohort.

Variable	≥80 Years (*n* = 32)	<80 Years (*n* = 32)	Effect Estimate (95% CI)	*p* Value
Operative time (min), median (IQR)	424.0 (335.5–553.0)	494.0 (447.3–590.5)	−63 (−130 to 21)	0.213
Blood loss (ml), median (IQR)	378.5 (213.5–613.8)	312.5 (181.3–626.3)	53 (−99 to 202)	0.204
Postoperative hospital stay(day), median (IQR)	29.0 (21.8–55.8)	22.0 (15.0–49.3)	6.5 (0 to 14)	0.368
Extent of lymphadenectomy, *n* (%)			—	0.079
Two-field	6 (18.8)	6 (18.8)		
Three-field	10 (31.2)	18 (56.2)		
Limited lymphadenectomy	16 (50.0)	8 (25.0)		
Number of dissected lymph nodes, median (IQR)	25.0 (14.0–34.3)	40.5 (23.5–68.8)	−15 (−30 to −4)	0.010
Number of metastatic lymph nodes, median (IQR)	1.0 (0.0–3.3)	1.0 (0.0–3.0)	—	0.831
Pathological stage, *n* (%)			—	0.603
0	3 (9.4)	0 (0.0)		
I	6 (18.8)	7 (21.9)		
II	7 (21.9)	8 (25.0)		
III	13 (40.6)	13 (40.6)		
IV	3 (9.4)	4 (12.5)		
R0 resection, *n* (%)	29 (90.6)	29 (90.6)	1.00 (0.12–8.10)	1.000
Clavien–Dindo grade ≥IIIa complications, *n* (%)	7 (21.9)	15 (46.9)	0.32 (0.09–1.06)	0.064
Pneumonia, *n* (%)	4 (12.5)	4 (12.5)	1.00 (0.17–5.94)	1.000
Recurrent laryngeal nerve palsy, *n* (%)	5 (15.6)	5 (15.6)	1.00 (0.20–4.90)	1.000
Anastomotic leakage, *n* (%)	6 (18.8)	11 (34.4)	0.45 (0.11–1.58)	0.257
30-day mortality, *n* (%)	0 (0.0)	1 (3.1)	—	1.000
90-day mortality, *n* (%)	0 (0.0)	2 (6.2)	—	0.492

IQR, interquartile range.

## Data Availability

All the data reported in this manuscript are available from the corresponding author upon reasonable request.
